# Considerations for sustainable influenza vaccine production in developing countries

**DOI:** 10.1016/j.vaccine.2016.08.056

**Published:** 2016-10-26

**Authors:** Claudia Nannei, Christopher Chadwick, Hiba Fatima, Shoshanna Goldin, Myriam Grubo, Alexandra Ganim

**Affiliations:** a Health Systems and Innovation Cluster, World Health Organization, CH-1211 Geneva 27, Switzerland; b Office of Pandemics and Emerging Threats, Office of Global Affairs, US Department of Health and Human Services, Washington, DC 20201, USA; c Duke Global Health Institute, Durham, NC 27710, USA; d Yale School of Public Health, New Haven, CT 06520, USA; e Centers for Disease Control and Prevention, Atlanta, GA 30333, USA

**Keywords:** Sustainability, Vaccine manufacturing, Pandemic influenza preparedness, Policy coherence

## Abstract

Through its Global Action Plan for Influenza Vaccines (GAP), the World Health Organization (WHO) in collaboration with the United States Department of Health and Human Services has produced a checklist to support policy-makers and influenza vaccine manufacturers in identifying key technological, political, financial, and logistical issues affecting the sustainability of influenza vaccine production. This checklist highlights actions in five key areas that are beneficial for establishing successful local vaccine manufacturing. These five areas comprise: (1) the policy environment and health-care systems; (2) surveillance systems and influenza evidence; (3) product development and manufacturing; (4) product approval and regulation; and (5) communication to support influenza vaccination. Incorporating the checklist into national vaccine production programmes has identified the policy gaps and next steps for countries involved in GAP’s Technology Transfer Initiative. Lessons learnt from country experiences provide context and insight that complement the checklist’s goal of simplifying the complexities of influenza prevention, preparedness, and vaccine manufacturing.

## Highlights

• Influenza vaccine.
• Vaccine manufacturing.
• Sustainability.
• Developing countries.
• Global Action Plan for Influenza vaccine.

## 1 Introduction

The World Health Organization (WHO) Global Action Plan for Influenza Vaccines (GAP) was developed in order to address challenges to sustainable influenza vaccine production and uptake in developing countries. Its aim is to increase equitable access to pandemic vaccines while contributing to international pandemic preparedness efforts. WHO directly supports 14 developing countries to establish or expand influenza vaccine manufacturing. This support is provided as part of a larger WHO mandate born within the Global Strategy and Plan of Action on Public Health, Innovation and Intellectual Property (GSPA-PHI) [1], approved in 2008 by Member States. Within the GSPA-PHI, the transfer of technology is a prominent feature to promote local production and improve access to medicines, vaccines, and diagnostics in developing countries. Transfer of technology principles align closely with the key components of sustainable local influenza vaccine production.

Within GAP’s objectives, there is a focus on increasing seasonal influenza vaccination coverage to stimulate global pandemic vaccine production capacity and to strengthen national regulatory competencies. However, the multi-sectorial nature of influenza vaccine manufacturing requires policy-makers and manufacturers to address a mix of technological, political, financial, and logistical issues that collectively affect sustainable production in developing countries [2].

GAP objectives also favour sustainable local vaccine production. Local vaccine production offers the following benefits [3]:

• better pandemic preparedness through development of local sources of vaccines;
• a more reliable supply of medical products, reducing the likelihood of distribution disruptions;
• more efficient supply chains in rural and poor areas and thus improved distribution times;
• potential cost savings as locally produced medical products, assuming the production scale is sufficiently large, are less expensive;
• higher quality products, better suited to local culture and conditions;
• stimulation of local innovation capacity and the development of human capital;
• development of export capacity for products, improving the national balance of payments; and
• increased employment generation and spill-over effects into other sectors of the economy.

**Fig. 1:**
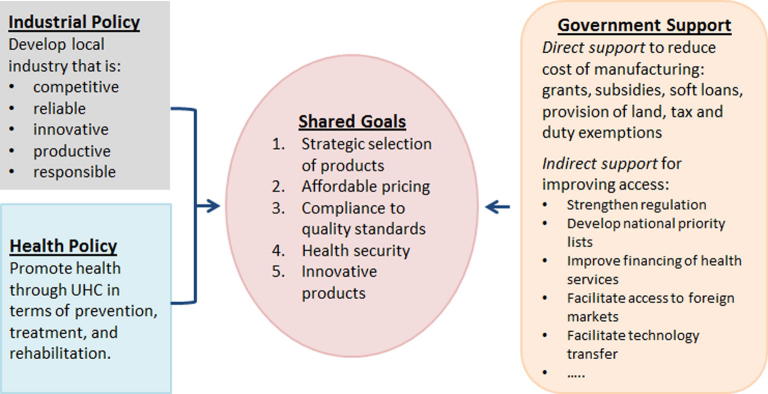
Multisectoral partnerships can improve local vaccine production, UHC; Universal health coverage.

Source: reference [4].

As shown in Fig. 1, health and industrial sectors can work together, with government support, to establish policies that contribute to their shared goals.

Local vaccine production and improved access to medical products can be stimulated by coordinated industrial, technology and health policies. Prioritization of local production can galvanize action targeting immediate barriers, such as lack of research and development capacities, resources, and expertise, which will result in broader impacts to the health, science, technology and industry fields. Governments are encouraged to examine these potential investments in light of the broader impact to other fields and as an opportunity to foster and drive industry and innovation locally. The return on investment must be substantial and sustainable for manufacturers to partner.

Sustainability also requires a coherent policy environment, reliable government procurement, product quality assurance, and market certainty. In addition to achieving public health objectives, there are strong economic and political drivers to establish and enhance national capacity to manufacture medical technologies.

## 2 Components of an enabling environment for sustainable local production of influenza vaccines

Over the last 10 years, through the implementation of GAP, WHO has distilled expertise and experiences into a checklist designed to support policy-makers and manufacturers in developing countries who are seeking to improve influenza vaccine sustainability. This checklist addresses the following areas: (1) the policy environment and health-care systems; (2) surveillance systems and influenza evidence; (3) product development and manufacturing; (4) product approval and regulation; and (5) communication to support influenza vaccination.

The checklist identifies, for each of the five specified areas, conditions that could increase the likelihood of sustainable local production and the use of influenza vaccines. These conditions are outlined below (see also Annex 1: Supplementary material).

### 2.1 Policy environment and health-care systems

National stakeholders develop policies to achieve their health objectives, based on recommendations developed by experts, such as Strategic Advisory Group expert (SAGE) on immunization, GAVI alliance, WHO, etc. International recommendations should be adapted to local specificities, while maintaining the global and inclusive approach to combat seasonal and pandemic influenza. Local vaccine production and improved access to medical products can be stimulated by coordinated industrial, technology and health policies.

#### 2.1.1 Health system policies

**Fig. 2:**
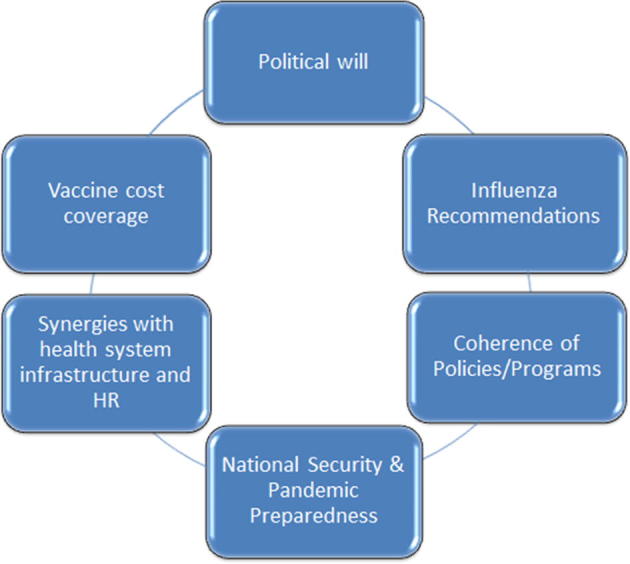
Health system policies: key components.

Six key interconnected desirable components of health system policies are listed below and represented visually in Fig. 2:

• Strong political will and buy-in are critical when establishing influenza as a national and/or regional priority. Establishing strong political will at leadership levels often results in increased resources, high-level public political commitment, cooperation to establish new policies or change existing ones, and enablement to navigate complex regulatory environments
• The use of international, regional, and national influenza recommendations to inform local policy development.
• Coherence among relevant national health policies and programmes ensures synergies between new and existing programmes.
• An established seasonal influenza vaccination programme can mitigate the effects of pandemic influenza events leading to stronger national security preparedness.
• A seasonal vaccination programme that leverages existing health system infrastructure and human resources to expand capacities in the routine health-care system.
• Inclusion of seasonal influenza vaccination in health insurance schemes or direct provision by the public health sector [5].

#### 2.1.2 Non-health system-based policies

**Fig. 3:**
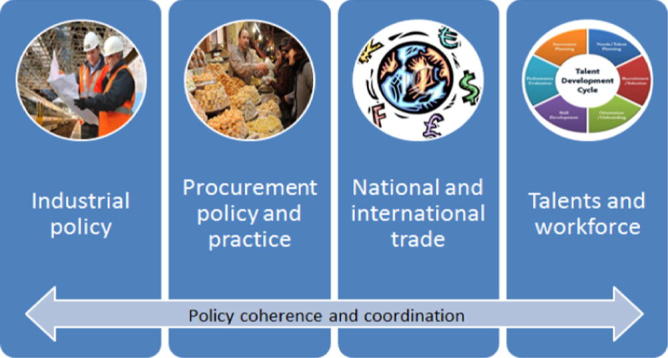
Beneficial non-health based policies.

In addition to health-focused policies, governments interested in developing local sustainable vaccine production are advised to coordinate relevant industrial, procurement, trade, and workforce policies. Fig. 3 provides a graphic depiction of these additional recommended policy avenues, which include:

• Interministerial coordination and strategic vision for industrial, health and economic development policies; this is crucial given the multi-sectorial space in which influenza vaccine manufacturing takes place.
• Policies that supports the development of good manufacturing practices. The policy environment should encourage scientific and academic institutions to conduct laboratory research as well as stimulate upstream and downstream research on biologicals and biomanufacturing.
• Relevant national and regional procurement and distribution policies to promote in-country production and sourcing of materials, in accordance with international agreements with third parties.
• Governments and manufacturers’ full awareness of the consequences that multilateral and bilateral trade agreements have on the economic and public health situation of the country with respect to commercialisation, such as the impact of removal of trade barriers for national manufacturers. The development of a skilled local workforce, through governmental incentives and education policies to prevent brain-drain, and partnerships between manufacturers and academia.

### 2.2 Surveillance systems and influenza evidence

**Fig. 4:**
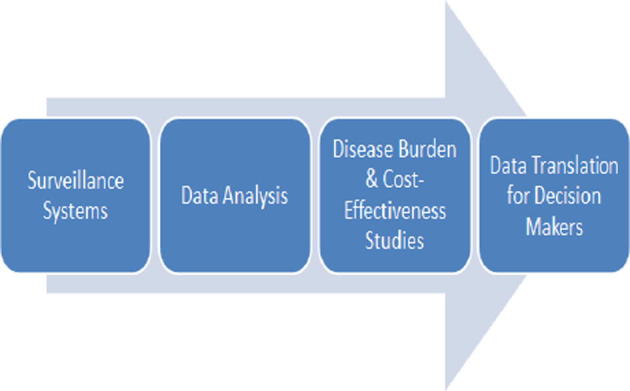
Data translation: from surveillance to policy.

Within any health system, the surveillance system has a critical role in providing relevant, accurate, and timely information for decision- and policy-making. Fig. 4 depicts the flow of information from the surveillance system and resulting data analysis to the subsequent disease burden and cost-effectiveness research. These results need to be framed within the larger political, economic, and geographic context for policy-makers. Conditions that favour effective evidence gathering include:

• A surveillance system that utilises all available resources, such as sentinel sites, population-based studies, insurance data, hospital and out-patient clinic, and regional data, to ensure regular burden of disease data collection.
• Clear definition of which data should be collected and which information systems should be used to accurately and appropriately analyse data that streamlines timely reporting to regional and international databases and dissemination of results.
• Investment in local burden of disease and cost-effectiveness studies to build evidence for influenza vaccination policies. If possible, consider probe^1^ studies to evaluate the vaccine-preventable burden of disease [6].
• Incorporation of morbidity and mortality data from neighbouring areas and various available proxies to understand the impact of seasonal influenza on the country population.
• Evidence generated by the data analysis should be framed by larger economic and geopolitical considerations for decision-makers, particularly in the context of global health security and pandemic preparedness.

### 2.3 Product development and manufacturing

Countries implementing policies to initiate local influenza vaccine production must consider the pipeline and manufacturing process. In this respect, the following would be considered advantageous:

• A solid business plan that considers the production environment and the marketplace where the product will be sold and used, the production costs and price of the product (which are influenced by the technology selected, the scale of production, other products produced in the facility, running costs, and infrastructure costs), the initial investment, and the time to market and regulatory pathway for the product.
• Reliable and stable supplies of utilities (e.g. water, electricity), with a focus on developing mechanisms that encourage self-sufficiency.
• Secure supply chain for all components, taking into account their costs, their foreseeable substitution, and their maintenance.
• Identification of available market niches to overcome constraints due to price of the product or size of the market.
• Strategies to mitigate business risks by considering seasonality, possible underutilisation of human resources and infrastructure, and additional products manufactured in the facility.
• Government and industry policies and programmes to bolster skilled workforce capacity and retention. Strong links and continuous dialogue with academic institutions support the availability of skills necessary to perform every step of the production process and ensure high managerial competencies.
• Realistic business and marketing strategies to identify return on investment, based on the potential size of the national, regional, and international markets.
• Full adherence to good manufacturing practice (GMP) and quality control procedures to guarantee a smoother production process and quality of the final product, thereby reducing the risk of costs linked to substandard quality steps or by-products.
• Strategically plan and design clinical trials and identify relevant partners for clinical trial administration. The product’s reference market is established by focusing on the target populations and reviewing the safety and effectiveness of the product. This also helps to create competitive advantages with the other manufacturers.
• Active engagement in advocacy associations and networks that sustain influenza vaccine manufacturing helps to raise awareness of issues faced by manufacturers in developing countries.
• Building in flexibility into various parts of the production process, including the sourcing of materials, increases pandemic preparedness. Also, the chosen technologies affect pandemic response time and the ramp-up capacity.

### 2.4 Product approval and regulation

In terms of creating an enabling regulatory environment for local influenza vaccine production the following considerations merit attention:

• The national regulatory authority (NRA) should be able to assess, license, control quality, and conduct surveillance of biological medical products. For countries producing vaccines, the NRA should be able to exercise the six recommended control functions^2^ in a competent and independent manner, backed up with enforcement.
• If the previous condition is met, the national manufacturer can aspire to meet WHO’s recommendation for United Nations prequalification of its product for better viability in local and international markets.
• With the emerging global market and the increasing number of novel products, the volume of biological medicinal products crossing national borders continues to rise. Participation in regional harmonisation efforts and integration of regulatory approvals plays a key role in facilitating the accuracy of regulatory work and international capacity building efforts.

### 2.5 Communication to support influenza vaccination

An effective national communication system comprises:

• A communication policy, plan, and strategy to support the development and implementation of influenza vaccination policies.
• Communication research, monitoring, and evaluation of objectives and agreed public health goals.
• Partnerships, stakeholders, and public engagement to implement the strategic objectives.
• Communication capacity building and training for all actors and stakeholders involved in influenza programmes.
• Knowledge translation and information communication technologies (ICTs) that support efficiency and effectiveness of the system and align them with the needs of 21st century information society.

## 3 Lessons learnt from the application of the checklist to national contexts

Over the last 4 years, WHO has conducted a series of assessments in developing countries^3^ to analyse national policy environments in which influenza vaccine manufacturing occurs. Through these experiences, several critical lessons and common findings have emerged that can be useful for other countries pursuing sustainable production and long-term availability of influenza vaccines. In particular, political awareness, financial accessibility of vaccination for targeted populations, and a strong NRA have been shown to provide a solid foundation for a coherent political and administrative environment.

The first notable lesson is the need for strong political support and government investment to develop a local product. After the 2009 influenza A (H1N1) pandemic, countries realised that they were ill-prepared and that national systems were too weak to protect their population. State involvement and support can take several forms: the development and updating of a pandemic influenza preparedness plan, financial commitment, and long-term supply agreements with manufacturers.

A second lesson learnt from countries’ experiences relates to access to health care. Studies show that a high rate of vaccination occurs when there is public or third-party reimbursement of vaccination, through government run health-care centres or insurance schemes [5], [7], [8]. When vaccinations are financially accessible to target populations, coverage rates increase substantially.

The existence of a strong NRA, operating in accordance with international and WHO standards, offers a strong support to the manufacturer during the product development and testing process. This regulatory presence also helps build confidence among the population that the vaccine is safe, effective, and of high quality. The NRA provides further assurance that the product will reach the population under reliable supply conditions, and the product will be monitored for safety throughout its lifespan.

Countries and manufacturers receiving technology for influenza vaccine production require skills and staff that are trained appropriately. From the manufacturers’ side, workers need to be trained to use the technology, and in parallel, awareness should be raised among regulators, administrative staff, surveillance system staff, and health-care workers to ensure that once the product is available, it is correctly administered. To this end, major investments in communication and training, especially of health-care workers, are paramount to ensure high levels of vaccination among at-risk populations [9].

Despite ample evidence of the disease burden imposed by influenza [10], [11] some countries have yet to conduct their own analyses of the socioeconomic impact of influenza. Strong evidence of the burden of disease will increase the understanding of the impact of the seasonal influenza virus and comorbidities and help target at-risk populations. Countries with well-developed surveillance systems in place are able to make informed decisions about the scale-up of influenza vaccine use. Probe studies are still underutilised, but could estimate the vaccine preventable disease incidence (VPDI)^4^ and establish causality of the disease [6].

WHO and countries developing influenza vaccine manufacturing and production capacity that have incorporated the checklist’s recommended targets have reported considerable successes. The use of the checklist has increased interagency communication and strategic planning that benefit both the short- and long-term viability of the influenza vaccine production.

## 4 Conclusion

The use of the checklist has proven useful in the identification of policy gaps and opportunities for better coherence of policies, as a tool for policy-makers and manufacturers to address the complexities of influenza prevention and preparedness and vaccine manufacturing. WHO country assessment reports include a compendium of policy options to strengthen sustainability of local production and use of influenza vaccines. These reports will be developed as a reference for countries willing to invest in influenza preparedness through local production, and will be adapted to the local context. By incorporating this checklist into a national vaccine production programmes, countries can cultivate comprehensive platforms for broader pandemic preparedness.

## Footnotes

1 Vaccine probe studies are randomised clinical trials used to understand the burden of a certain disease and how vaccine use can potentially decrease this burden. They can also be used to establish causation between a specific pathogen and disease[6].

2 License of manufacturers and product regulation; surveillance of vaccine field performance (post-marketing surveillance); system of lot release; laboratory access (use of laboratory when needed); regular inspections for GMP; clinical trial approval (evaluation of clinical performance).

3 The exercise has been applied in seven countries: Brazil, Indonesia, Mexico, Morocco, South Africa, Thailand, and Viet Nam.

4 The vaccine preventable disease incidence (VPDI) is the difference in the incidence of disease between vaccinated and unvaccinated people.
