# Supplementary figures and images for: Considerations for sustainable influenza vaccine production in developing countries
Source: Vaccine. 2016 Oct 26;34(45):5425–9. doi: 10.1016/j.vaccine.2016.08.056 (PMC5359512; doi:10.1016/j.vaccine.2016.08.056)

Annex 1: Supplementary material (version2)


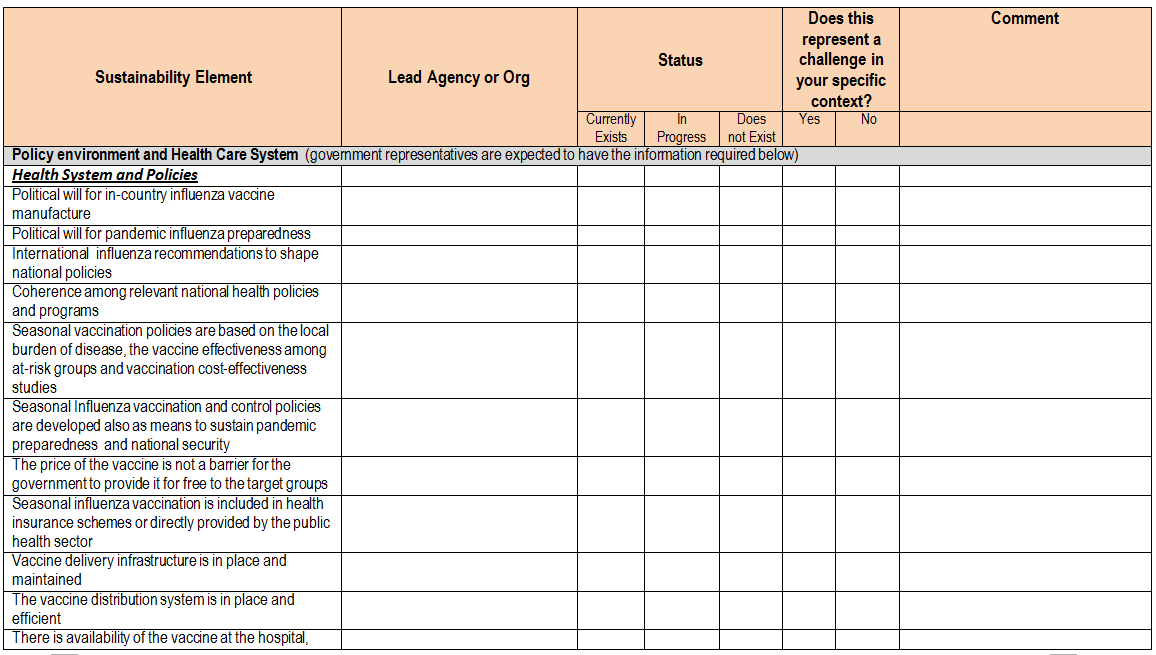

Supplement: Supplementary data 1 [file mmc1.docx]
